# X-Linked and Autosomal Recessive Alport Syndrome: Pathogenic Variant Features and Further Genotype-Phenotype Correlations

**DOI:** 10.1371/journal.pone.0161802

**Published:** 2016-09-14

**Authors:** Judith Savige, Helen Storey, Hae Il Cheong, Hee Gyung Kang, Eujin Park, Pascale Hilbert, Anton Persikov, Carmen Torres-Fernandez, Elisabet Ars, Roser Torra, Jens Michael Hertz, Mads Thomassen, Lev Shagam, Dongmao Wang, Yanyan Wang, Frances Flinter, Mato Nagel

**Affiliations:** 1 The University of Melbourne, Melbourne Health and Northern Health, Melbourne, Australia; 2 Molecular Genetics Laboratory, Guy’s and St Thomas’ Hospital, London, United Kingdom; 3 Research Coordination Center for Rare Diseases, Seoul National University Hospital, Seoul, Korea; 4 Institut de Pathologie et Genetique, Department of Molecular Biology, Gosselles, Belgium; 5 Lewis-Sigler Institute for Integrative Genomics, Princeton University, Princeton, New Jersey, United States of America; 6 Molecular Genetics Centre GENETAQ, Malaga, Spain; 7 Molecular Biology Laboratory and Department of Nephrology, REDINREN, Fundacio Puigvert, Universitat Autonoma de Barcelona, Barcelona, Spain; 8 Department of Clinical Genetics, Odense University Hospital, Odense, Denmark; 9 Institute of Pediatrics, Pirogov Russian Medical University, Moscow, Russia; 10 Department of Genetics, Guy’s and St Thomas’ Hospital, London, United Kingdom; 11 Centre for Nephrology and Metabolic Medicine, Weisswasser D-02943, Germany; National Cancer Institute, UNITED STATES

## Abstract

Alport syndrome results from mutations in the *COL4A5* (X-linked) or *COL4A3*/*COL4A4* (recessive) genes. This study examined 754 previously- unpublished variants in these genes from individuals referred for genetic testing in 12 accredited diagnostic laboratories worldwide, in addition to all published *COL4A5*, *COL4A3* and *COL4A4* variants in the LOVD databases. It also determined genotype-phenotype correlations for variants where clinical data were available. Individuals were referred for genetic testing where Alport syndrome was suspected clinically or on biopsy (renal failure, hearing loss, retinopathy, lamellated glomerular basement membrane), variant pathogenicity was assessed using currently-accepted criteria, and variants were examined for gene location, and age at renal failure onset. Results were compared using Fisher’s exact test (DNA Stata). Altogether 754 new DNA variants were identified, an increase of 25%, predominantly in people of European background. Of the 1168 *COL4A5* variants, 504 (43%) were missense mutations, 273 (23%) splicing variants, 73 (6%) nonsense mutations, 169 (14%) short deletions and 76 (7%) complex or large deletions. Only 135 of the 432 Gly residues in the collagenous sequence were substituted (31%), which means that fewer than 10% of all possible variants have been identified. Both missense and nonsense mutations in *COL4A5* were not randomly distributed but more common at the 70 CpG sequences (p<10^−41^ and p<0.001 respectively). Gly>Ala substitutions were underrepresented in all three genes (p< 0.0001) probably because of an association with a milder phenotype. The average age at end-stage renal failure was the same for all mutations in *COL4A5* (24.4 ±7.8 years), *COL4A3* (23.3 ± 9.3) and *COL4A4* (25.4 ± 10.3) (*COL4A5* and *COL4A3*, p = 0.45; *COL4A5* and *COL4A4*, p = 0.55; *COL4A3* and *COL4A4*, p = 0.41). For *COL4A5*, renal failure occurred sooner with non-missense than missense variants (p<0.01). For the *COL4A3* and *COL4A4* genes, age at renal failure occurred sooner with two non-missense variants (p = 0.08, and p = 0.01 respectively). Thus DNA variant characteristics that predict age at renal failure appeared to be the same for all three Alport genes. Founder mutations (with the pathogenic variant in at least 5 apparently- unrelated individuals) were not necessarily associated with a milder phenotype. This study illustrates the benefits when routine diagnostic laboratories share and analyse their data.

## Introduction

Alport syndrome is the commonest cause of inherited renal failure after polycystic kidney disease [1]. It affects at least one in 10,000 individuals and is characterised by progressive kidney failure, hearing loss, and ocular abnormalities [2]. Inheritance is X-linked with *COL4A5* mutations [3] in 85% of cases and autosomal recessive with *COL4A3* or *COL4A4* mutations in most of the others [4,5]. Individuals with heterozygous *COL4A3* or *COL4A4* mutations usually have Thin basement membrane nephropathy with normal renal function [6,7,8] but some develop renal impairment [9].

The clinical features in Alport syndrome are explained because the *COL4A5* and *COL4A3/COL4A4* genes code for the collagen IV α5 chain, α3 and α4 chains which form a heterotrimer in the basement membranes of the glomerulus, cochlea and eye [10] (Table 1). Each collagen IV chain has non-collagenous amino and carboxy termini, and an intermediate collagenous domain with Gly-X-Y, with multiple short non-collagenous interruptions [10]. Glycine is found at each third residue in the collagen sequence and is critical for triple helix formation.

**Table 1:** Characteristics of *COL4A5*, *COL4A3* and *COL4A4* genes and the corresponding proteins.

	*COL4A5*^*1*^	*COL4A3*^*2*^	*COL4A4*
Gene name	NP_033380.2	NP_000091.4	NP_000092.4
Chromosomal location	Xq22	2q35-37	2q35-37
Family	*COL4A1-like	*COL4A1-like	*COL4A2-like
Number of exons	53	52	48
Size	257,623 bp	150,228 bp	159,344 bp
Total number of amino acids in mature protein	1665	1642	1652
Signal peptide	26	28	38
Amino terminus	15	14	23
Collagenous domain (residues)	1301	1268	1284
Number of non-collagenous interrruptions	22	23	26
Carboxy terminus (residues)	229	232	231

*COL4A–like or *COL4A2*-like genes represent families that have arisen from common progenitors, *COL4A1* or *COL4A2*, and thus have structural similarities

The ‘Expert guidelines on the diagnosis and management of Alport syndrome’ [2] recommend that all individuals with likely Alport syndrome should undergo genetic testing, to confirm the diagnosis and mode of inheritance, and predict the age at renal failure. The Guidelines also recommend cases of suspected Thin basement membrane nephropathy undergo genetic testing when it is important to exclude X-linked Alport syndrome [2].

Mutations in the *COL4A5*, *COL4A3* and *COL4A4* genes are mainly nonsense or missense. Genotype-phenotype correlations have only been characterised for X-linked disease [11,12,13]. Nonsense mutations are typically associated with early onset renal failure (before 30), lenticonus and central retinopathy. Deletions and insertions often cause a frameshift, and downstream nonsense variant. Many missense mutations result in a milder phenotype with later onset renal failure without ocular features [11,12,13,14].

The commonest method used currently for mutation detection is whole exome sequencing (WES). The widespread adoption of WES together with the Exome Aggregation Consortium (ExAc collaboration, http://exac.broadinstitute.org/, the ‘1000 genomes’, http://1000genomes.org/ and Hapmap, https://hapmap.ncbi.nlm.nih.gov/, projects), have greatly increased the number of reported pathogenic and normal DNA variants [15].

Collecting all pathogenic and normal variants for each disease-related gene in a publicly-accessible variant database avoids the duplication involved in assessing pathogenicity, and improves the speed and accuracy of testing while limiting cost.

The present study has reviewed known variants in the Alport genes, including 754 recently-submitted from members of the Alport mutation database Consortium to the publicly-accessible LOVD databases, with the aims of further characterising DNA variants, and genotype-phenotype correlations. The LOVD databases continue to be updated.

## Methods

### Assembly of the variants

DNA variants were extracted from the literature, from other databases (eg ARUP) or from previously-unpublished deidentified submissions direct to the LOVD *COL4A5*, *COL4A3* and *COL4A4* database from contributing members of the International Alport Mutation Database Consortium in the UK and Europe, Korea, the US and Australia. Genetic testing from laboratories run by Consortium members was performed after written informed consent had been obtained by the treating clinicians. Submission of variants to the databases was undertaken with IRB approval where this was required (Northern Health HREC, IRB of Seoul National University, CEIC Fundacio Puigvert, Veltishev Pediatric Clinical Research Institute).

In general, individuals had been referred for genetic testing where Alport syndrome was suspected clinically or on renal biopsy (renal failure, hearing loss, retinopathy, lamellated glomerular basement membrane). Clinical data (age, gender, diagnosis and age at onset of renal failure, and, where known, hearing loss and ocular abnormalities) were obtained in a manner conforming with local IRB ethical guidelines according to the Declaration of Helsinki. The testing laboratory deidentified information before submission to the databases.

Variants had been collected by individual laboratories over a ten year period and mutation detection methods varied during this time. All laboratories had used Sanger sequencing at some stage, few had used MLPA, but recently most had used whole exome sequencing. Most diagnostic laboratories confirmed any variant found with WES using Sanger sequencing. All laboratories were accredited by their national board but there was no uniformity in how they confirmed variant pathogenicity. This was most often with consulting the LOVD, and recently, the ExAc databases, and sometimes using an online tool such as Alamut.

Variants were described according to the reference transcripts of *COL4A5* (the longer form found in the kidney, LRG_232t2, NM_033380.2), *COL4A3* (LRG_230t1, NM_000091.4) and *COL4A4* (LRG_231t1, NM_000092.4), and the nomenclature guidelines of the Human Genome Variation Society (HGVS) [16]. Variant descriptions were confirmed with the Mutalyzer programme (http://mutalyzer.nl/) [17]. Pathogenicity assessments were as published or as provided by the submitting laboratory. In general these were based on the HGVS definitions [18]. Variants were not checked independently with any algorithms such as the Human Splicing Finder because their efficacy and accuracy is not confirmed in the genes of interest. Our definition for splicing mutations included the 10 intronic nucleotides immediately adjacent to the exon-intron boundaries. Deep intronic variants were only included where the submitting laboratory believed them to be disease-causing.

Variants, demographic and clinical data were added to the LOVD databases that included all published *COL4A5*, *COL4A3* and *COL4A4* variants. Disease-associated variants already recorded in the database but from apparently unrelated families were included to identify the prevalence of Alport syndrome and any common or founder mutations. Mode of inheritance, gender and clinical features were noted where available.

### Analysis of the LOVD *COL4A5*, *COL4A3* and *COL4A4* databases

Different variant types (deletions, insertions, short deletions, arbitrarily chosen as < than 26 bp, and splicing variants) direct or indirect nonsense mutations, missense variants (resulting in Gly or non-Gly substitutions, pathogenic or not, and synonymous changes) were counted. Each variant was included once only in the analysis.

Large deletions were mapped against the collagen sequence. Missense variants corrected for exon size were compared between exons to determine any increase in variants or gaps.

In addition, *COL4A5* missense mutations were tested for a preferential occurrence in CpG-associated codons [19] (especially involving the first G of Gly) using an in-house Python script (www.python.org). CpG sites have an increased tendency for methylcytosine to spontaneously deaminate to thymine. CG>TG and the complementary changes have a 10 times higher mutation rate than normal.

The prevalence of Gly substitutions with each of 8 other amino acids or a stop codon was compared with the expected distribution determined from collagen codon preferences [20].

### Age at onset of renal failure

The nature of mutations was correlated with early and late onset renal failure, using the criteria already identified for *COL4A5 ‘*severe’ variants (copy number variants, frameshifts, nonsense and splice site mutations). Only one age was used for the variant in each family, and for *COL4A5* variants, the ages at renal failure in males and females were studied separately. Where several members of the same family or unrelated individuals with the same mutation were described, the youngest age of onset of renal failure was recorded. For *COL4A3* and *COL4A4*, the age at end-stage renal failure in males and females were combined, and the number of severe variants recorded as none, one or two.

### Statistical analysis

Results were expressed as mean and SD, and results compared using Fisher’s exact test (DNA Stata). A p value of less than 0.05 was considered significant, and a p values less than 0.10, a trend.

## Results

Twelve laboratories involved worldwide in mutation testing for Alport syndrome collaborated to submit 754 novel variants (504 *COL4A5*, 133 *COL4A3*, 117 *COL4A4* variants) to the LOVD databases. These contributions increased the total number of variants in the databases by 25% (754/2959), to 1168 unique variants for *COL4A5* (1951 total), 266 for *COL4A*3 (518 total) and 268 for *COL4A4* (490 total) (Table 2). Most variants were pathogenic, or of unknown significance, from individuals with Alport syndrome or Thin basement membrane nephropathy. Most were from Europeans with *COL4A5* variants, or were from Chinese (72 pathogenic, 12 normal), African (10, 8 normal), Hispanic (7 pathogenic) or Indian (2 normal) people.

**Table 2:** Frequency of variants in *COL4A5*, *COL4A3* and *COL4A4* genes in LOVD databases.

	*COL4A5* LOVD	*COL4A3* LOVD	*COL4A4* LOVD
Total number of variants	1168 unique (1951 total)	266 unique (518 total)	268 unique (490 total)
Rearrangements or deletions, copy number variants	76 (7%)	0	3 (1%)
Duplications	13 (1%)	3 (1%)	4 (1%)
Insertions	55 (5%)	8 (3%)	8 (3%)
Deletions (1-26bp)	169 (14%)	31 (12%)	32 (12%)
Indels (with both insertions and deletions)	16 (1%)	3 (1%)	2 (1%)
Splicing variants	273 (152+ and 121-)* (23%)	25 (15+ and 10-)* (9%)	52 (25+ and 27-)* (19%)
Nonsense codons (direct)	73 (6%)	13 (5%)	13 (5%)
Total potential nonsense mutations (direct and downstream)	329 + 73 = 402 (34%)	45 + 13 = 47 (18%)	13 + 49 = 62 (23%)
Missense substitutions	504 (43%)	136 (51%)	107 (40%)
• Gly	391 (33%)	66 (25%)	46 (17%)
• Non-Gly	113 (10%)	70 (26%)	61 (23%)
Non-pathogenic changes	24	78	65
• Synonymous	11	44	28
• Non-Gly missense	7	28	14
• Deep intronic	6	6	23

* For splicing variants, + indicates that the variant is located within the intron immediately adjacent to the 3’ end of the exon; and—indicates that the exon is within the intron immediately adjacent to the 5’ end of the exon.

### DNA variants in *COL4A5*

The 1168 *COL4A5* unique variants included 76 complex mutations or deletions of more than 26 nucleotides (7%), 55 insertions (5%), 13 duplications (1%), 169 short deletions (with fewer than 26 nucleotides, 14%), and 16 indels (1%), 73 nonsense mutations (6%), 504 missense variants (43%) and 273 (23%) splicing variants (Table 2). Uncommonly two pathogenic variants were found in the same individual (11/1168, 1%).

#### Large deletions/indels/rearrangements

There were 25 large deletions. Seven originated in *COL4A6* and terminated in exon 1 or the nearby intron of *COL4A5*. Eight large deletions originated in exons 1–4. Only 9 deletions (fewer than half) had their origins after exon 4, and six at exons 37 to 41.

#### Direct nonsense mutations

There were 73 (6%) direct nonsense mutations but the insertions/deletions and duplications that result in a frameshift meant that there were potentially 402 variants producing a downstream nonsense change (34%). In addition, 17 direct stop codons resulted from Gly substitutions (4%) which matched the expected prevalence (4%) (Table 3).

**Table 3:** Observed versus expected likelihood of Glycine substitutions in the *COL4A5*, *COL4A3* and *COL4A4* genes in Alport syndrome and Thin basement membrane nephropathy. Observed number from the LOVD databases; expected number derived from data for collagen I [20].

	Ala	Ser	Cys	Arg	Val	Glu	Asp	Trp	Stop
Expected (based on residue frequency and collagen coding preferences)	13%	18%	6%	26%	10%	14%	14%	1%	4%
*COL4A5* observed (n = 391)	15 (4%) (p less than 0.0001)	45 (11%) (p = 0.02)	19 (5%)	86 (22%)	64 (16%)	56 (14%)	68 (17%)	3 (1%)	17 (4%)
*COL4A3* observed (n = 66)	0 (0%)	8 (12%)	6 (9%)	23 (35%)	6 (9%)	13 (20%)	8 (12%)	1 (1%)	1 (1%)
*COL4A4* observed (n = 46)	1 (2%) (p = 0.06)	7 (15%)	3 (7%)	15 (33%)	4 (9%)	11 (24%)	3 (7%)	1 (2%)	1 (2%)

p- not significant for all other comparisons

#### Missense mutations

There were 504 missense variants (43%) of which 391 (33%) were Gly substitutions, and 113 (10%) non-Gly substitutions. Thus Gly in *COL4A5* was substituted three times more often than other amino acids. Glycine can be substituted nine ways (Table 3), and the substitutions with each residue occurred as often as expected except for Ala which was underrepresented (4% compared with 13% expected). Ala is the least destabilising amino acid, and results in a milder clinical phenotype, which may have been overlooked clinically (p<0.0001)[20].

Of the 113 non-Gly substitutions, the most common replacements were of Arg, Asp, Glu, Ser (26, 9.5%), Val (10, 4%) or Ala (7, 2.6%), to Gly.

Only 135 of the 432 Gly in the collagenous sequence were substituted (31%). This was comparable with *COL1A1* where fewer than 10% of all variants were calculated to be known (Marini, 2007). Most substitutions were with one amino acid only.

**Fig 1:**
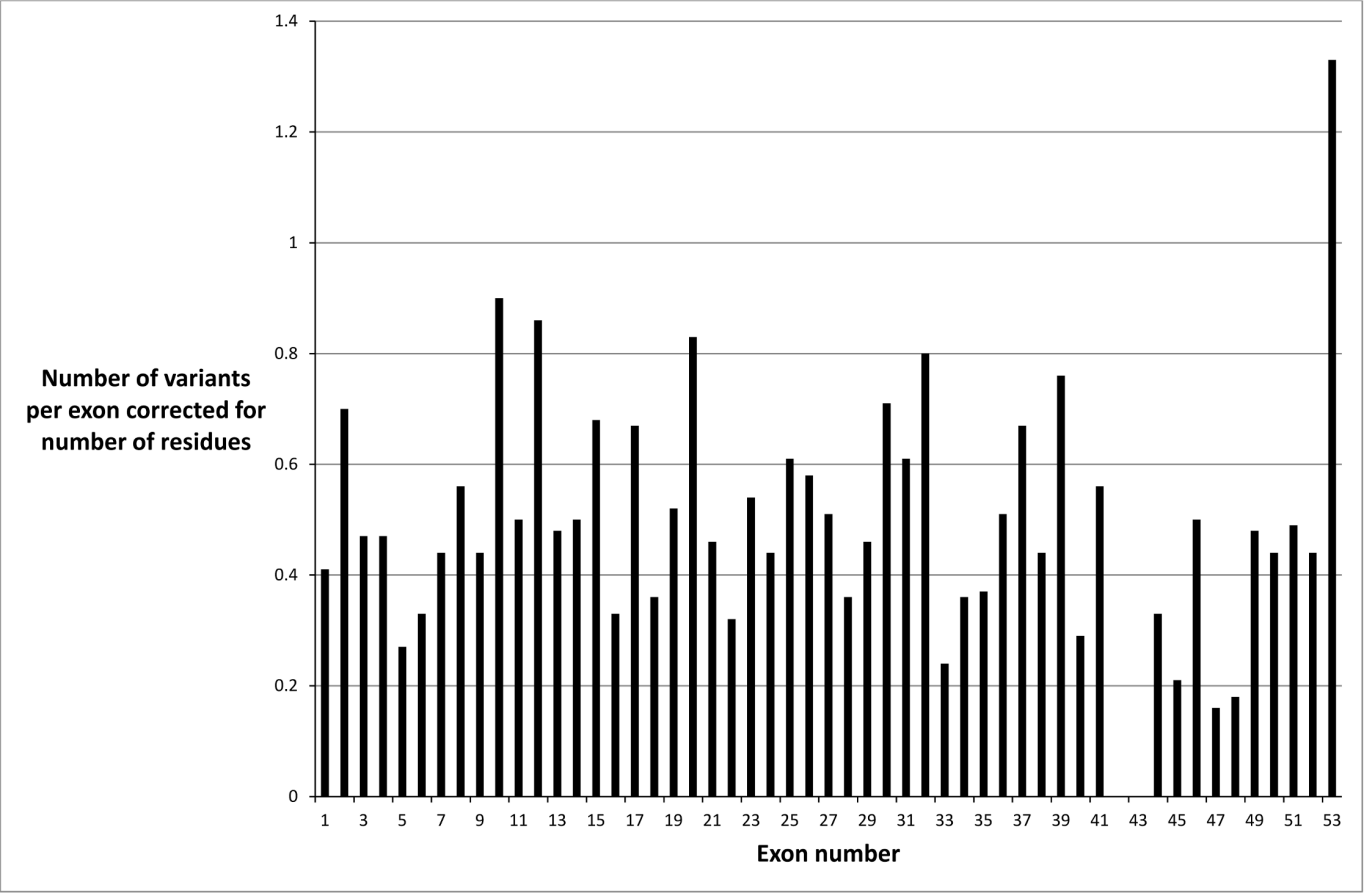
Distribution of missense variants in the *COL4A5* gene in each exon corrected for exon size and demonstrating a non-uniform distribution.

Missense variants were not distributed evenly throughout the *COL4A5* gene when exon size was taken into account (Fig 1). There were fewer mutations in exons 47 and 48, which suggested a milder phenotype or an ascertainment bias. It was unlikely that the fewer mutations were due to an embryonic lethal phenotype because the collagen IV α3α4α5 network is mainly expressed after infancy. However exons 47 and 48 correspond to the collagen IV α5 chain before the carboxy terminus, where there is a ‘gap’ for entry of the cleavage enzymes, and the major epithelial binding site on the parallel IV alpha3 chain [21]. Mutations were also underrepresented in exons 42 and 43 probably because these exons are small and not routinely sequenced.

#### CpG site mutations

There were 70 CpG sites among the 5076 nucleotide positions in *COL4A5*. Of all the mutations causing Alport syndrome, 89 were present at CpG sites which was more often than by chance (p less than10^- 41^ by Fisher’s exact test). Nonsense mutations were also observed more often at CpG sites. Of the 89 mutations at 70 CpG sites, 27 were nonsense changes at 5 sites (p less than 0.001 by Fisher’s exact test).

Gly substitutions within the collagenous domains were also overrepresented in *COL4A5* mutations. Of the 491 missense mutations affecting the 1347 amino acid positions in collagen IV α5 chain, 435 (89%) were substitutions of one of the 449 collagenous domain Gly residues (p<0.001). Similarly, at the CpG sites, 46 missense mutations were observed at 43 CpG sites within the collagenous domains, with 39 resulting in Gly substitutions at 12 Gly positions (p less than 10^−7^ by Fisher’s exact test).

#### Splice site mutations

There were 273 splicing variants (23% of total number) with a variant within 10 nucleotides of the intron-exon boundary that was possibly pathogenic. At least one variant was identified for each intron, and there were 152 + (at the 3’ end of the exon) and 121 –(at the 5’ end of the exon) splice variants.

#### Founder mutations

Two hundred and sixty-five pathogenic variants (23%) were recorded more than once, but only 12 reported at least five times in apparently unrelated families (Table 4). Three were biochemically ‘severe’ mutations being a duplication or nonsense variant. A further three were Gly substitutions. Six resulted in renal failure at less than 30 years of age, and five in a milder phenotype with later onset renal failure.

**Table 4:** Common Founder mutations in the *COL4A5*, *COL4A3* and *COL4A4* genes.

	Variant	Number of reports/families	Ethnicity	Clinical features, Age at onset of renal failure	Reference
*COL4A5*	Tandem duplication of 35 exons	>160 individuals	French Polynesian	ESRF at 31 years, lenticonus	[28]
	p.Gly325Arg	Reported 9 times	France, European	ESRF at 22–76 years, hearing loss	[29]
	p.Arg373*	Reported 6 times	Italian	ESRF at 15 years, hearing loss	[30]
	p.Gly624Asp	Reported at least 5 times	European	ESRF late	[31,32]
	p.Gly869Arg	Reported 12 times	Italian	ESRF at 10 years	[30]
	p.Ser916Gly	Reported 5 times	US	Not available	[33]
	p.Gly953Val	Reported 6 times	France	ESRF at 15 years, hearing loss	[34]
	p.Gly1030Ser	Reported 5 times	N European, US	ESRF at 37 years, hearing loss	[31]
	p.Arg1569Gln	Reported 7 times	US, France	ESRF at 29 years, hearing loss	[34,35]
	p.Leu1655Arg	Reported in 9 families	Western US	ESRF at 40 years, hearing loss	[36]
	p.Arg1683Gln	Reported 5 times	Ashkanazi-American	ESRF 40–62 years	[37]
	p. Arg1683*	Reported 6 times	British	ESRF at 27 years, hearing loss	[38]
*COL4A3*	c.40-63del	One in 183 Ashkenazi	Askhkenazi-American	ARAS	[39]
	p.Gly43Arg	Reported 5 times	European, US	TBMN, AR AS	[40]
	p.Glu162Gly	Reported 8 times	European, US	TBMN, AR AS	[40]
	p.Gly695Arg	Reported 5 times	British	Haematuria, TBMN	[41]
	p.Gly871Cys	6 families	Cypriot	Haematuria, TBMN and FSGS	[42,43]
	p.Gly1334Glu	4 families	Cypriot	TBMN, FSGS	[42,43]
	p. Gln1495Arg	Reported 5 times	European, British, Cypriot	AR AS, TBMN	[44]
	p. 13_22 del LPLLLVLL	1:183 of Ashkanazi	American Ashkenazi	AR AS	[39]
*COL4A4*	p.Gly545Ala	Reported 11 times	Turkish, German	TBMN, AR AS	[45]
	c.2384-5T>C	Reported 6 times	Italian	TBMN	[44]
	p.A880Hisfs69*	Reported 5 times	Australian	AR AS	[46]
	p.Gly960Arg	Reported 6 times	Spanish	TBMN	[47]
	p.Ser969*	Reported 14 times	British	AR AS with early onset ESRF; TBMN	[48]

*stop codon; ESRF–end-stage renal failure, FSGS–focal segmental glomerulosclerosis, AR AS–autosomal recessive Alport syndrome, TBMN–thin basement membrane nephropathy

Some of these mutations will have arisen from independent events and others from a single individual, but the distinction can only be made with haplotype analysis. These mutations allow us to examine the consistency of age of onset of renal failure and the effect of modifying genes. More founder mutations are likely but laboratories may not have not reported them because of their ‘lack of novelty’.

#### Genotype-phenotype correlation

Clinical data were most complete for age at end-stage renal failure. Formal hearing and ophthalmic examinations were rarely reported to the testing laboratory.

Reported mutations were associated more often with a severe phenotype. The age at onset of renal failure was available for 237 *COL4A5* mutations (12%) and the mean age was 24.3 years (Table 5). More mutations (162, 68%) were associated with end-stage renal failure before the age of 30 years, than later (75, 32%).

**Table 5:** Age at end-stage renal failure and severity of mutations with *COL4A5*, *COL4A3* and *COL4A4* variants.

	*COL4A5*	*COL4A3*	*COL4A4*
Total number with end-stage renal failure and all causative mutations identified	237	75	48
Age at renal failure for all patients with mutations in this gene (mean, SD, years)	24.4, 7.8 (23.4 to 25.4) (n = 237) (*COL4A5* and *COL4A3*, p = 0.45; *COL4A5* and *COL4A4*, p = 0.55)	23.3, 9.3 (20.1 to 26.5) (n = 35) *(COL4A3* and *COL4A4*, p = 0.41).	25.4, 10.3 (21.3 to 29.6) (n = 26)
End-stage renal failure at < 30 years	162 (68%)		
Number of homozygous mutations	N/A	16 (21%)	15 (31%)
Number of compound heterozygous mutations	N/A	59 (79%)	33 (69%)
	Age at onset of renal failure		
No severe mutation (mean, SD, years)	26.7, 8.1 (n = 106)	24.0, 6.9 (n = 8)	26.6, 5.0 (n = 9)
One severe mutation (mean, SD, years)	22.5, 7.0 (n = 131) (one compared with none, p less than 0.01)	20.8, 5.1 (n = 8)	25.5, 7.8 (n = 4)
Two severe mutations (mean, SD, years)	Not applicable	17.6, 8.5 (n = 14) (two compared with none, p = 0.08)	21.1, 3.6 (n = 10) (two compared with none, p = 0.01)
Direct nonsense mutation	21.4, 6.4 (16.9 to 25.9) (n = 12)	17.9, 5.5, (13.3 to 22.5) (n = 8)	

‘Severe’ mutations were large rearrangements, deletions, insertions or other changes resulting in a nonsense codon.

A severe mutation (rearrangement, large deletion, insertion/deletion, or nonsense change) was associated with a younger average age at onset of renal failure of 22.5 ± 7.0 years (n = 131) than in those without a severe mutation (26.7 ± 8.1 years, n = 106, p less than 0.01).

A direct nonsense mutation, that is where a codon was replaced by a stop signal, was associated with a younger average age at onset of renal failure of 21.4 ± 6.4 years than with other mutations types (p = 0.03).

There was no difference in the age at onset of renal failure in individuals with Gly substitutions with Arg, Glu or Asp compared with other amino acids, nor with non-Gly substitutions (p all greater than 0.05).

*COL4A5* mutations were reported in 68 females. Nine of the 23 (39%) who developed renal failure had a direct or indirect nonsense mutation or rearrangement or insertion/deletion, in contrast to 9 of the 45 without renal failure (20%, p = 0.14).

### DNA variants in *COL4A3* and *COL4A4*

Many fewer DNA variants have been described for the *COL4A3* and *COL4A4* genes, presumably because autosomal recessive Alport syndrome is less common, and genetic testing is performed infrequently for Thin basement membrane nephropathy.

DNA variants in the LOVD databases affected the *COL4A3* and *COL4A4* genes equally frequently (Table 2). The *COL4A3* database had 266 unique variants, and *COL4A4* had 268. Compound heterozygous variants were more common than homozygous variants for both *COL4A3* and *COL4A4* (69% and 59% respectively) suggesting lesser consanguinity.

#### Large deletions/indels/rearrangements

The proportions of duplications, insertions, short deletions and other indels were similar to those found in *COL4A5*.

#### Missense

Glycine substitutions were relatively less abundant than for *COL4A5* (Table 2). This was unexpected since they are the most common change in X-linked Alport syndrome and other collagen diseases such as osteogenesis imperfecta [20]. The explanation may be that milder disease with heterozygous Gly substitutions was overlooked. Gly to Ala or Ser substitutions were again underrepresented for the *COL4A3* and *COL4A4* genes.

Too few missense variants were found to determine any mutational hot spots. Non-Gly substitutions were more common than in *COL4A5*, but their pathogenic significance was often not clear.

#### Nonsense

The % of direct nonsense mutations was similar to *COL4A5* but the total % (of direct and potential indirect nonsense mutations) was fewer because fewer large insertions/deletions were reported.

#### Splicing

Very few potential splicing mutations were identified within the first 10 nucleotides of the intron-exon boundaries.

#### Founder mutations

Founder mutations were reported in both *COL4A3* and *COL4A4* in autosomal recessive Alport syndrome and Thin basement membrane nephropathy (Table 5). For example, the S969X variant in *COL4A4* was the commonest variant in British populations, and other variants were reported from Cyprus.

#### Genotype-phenotype correlations

Clinical features were compared between individuals with autosomal recessive Alport syndrome caused by *COL4A3* or *COL4A4* pathogenic mutations and those with X-linked Alport syndrome and *COL4A5* mutations. The age at onset of renal failure was known for some homozygous or compound heterozygous *COL4A3* (n = 35) and *COL4A4* (n = 25) pathogenic mutations where both variants were known. Overall the mean age at onset of end-stage renal failure was not different for individuals with *COL4A3* (23.2 + 9.3, years, 95% CI 20.1 to 26.5) or *COL4A4* mutations (mean 25.4 ±10.3 years, 95% CI 21.3 to 29.6) compared with *COL4A5* mutations (24.4 ± 7.8 years, 95% CI 23.4 to 25.4, n = 237) (Table 4).

The age at onset of renal failure was also the same for homozygous and for compound heterozygous *COL4A3* and *COL4A4* mutations (21.5 ± 7.2, 95%CI 17.2–25.9, n = 13; and 21.8 ± 7.0, 95% CI 19.8 to 23.7, n = 52).

Subjects with two severe mutations (direct or indirect nonsense mutations, insertions/deletions) developed end-stage renal failure at a younger age than those with none (Table 4). Individuals with at least one direct nonsense *COL4A3* or *COL4A4* mutation also developed renal failure at a younger age than those with none. Thus, the age at onset of renal failure in autosomal recessive Alport syndrome depended on mutation severity using the same criteria as for *COL4A5* mutations [11,12]. However this study did not demonstrate any difference in the age at end-stage renal failure due to Gly substitutions with Arg, Glu or Asp compared with other substitutions for *COL4A3* and *COL4A4*.

Twenty *COL4A3* or *COL4A4* mutations were identified in the LOVD databases in individuals diagnosed clinically with autosomal dominant Alport syndrome. Seven only had developed end-stage renal failure, at a mean age of 42 ± 15.1 years (range 20–60). The others either did not have renal failure or the age at onset was not known.

### Biallelic and digenic mutations

There are occasional reports of individuals with two *COL4A5* mutations [22,23], and others with autosomal recessive Alport syndrome and mutations in both *COL4A3* and *COL4A4* with a later age at onset of renal failure [15,24,25].

## Discussion

This study confirmed that *COL4A5*, and *COL4A3* and *COL4A4* mutations in Alport syndrome all resulted in end-stage renal failure at the same age. Other studies have found that *COL4A5* deletions, insertions, and nonsense variants, are associated with early onset renal failure [11,12,13]. This study confirmed that two *COL4A3* or *COL4A4* mutations resulted in earlier onset renal failure than one or no severe mutations. Nonsense *COL4A3* and *COL4A4* mutations were also associated with a younger age at renal failure. Cohort studies indicate that hearing loss is common with all mutation types, and that the same mutation types that cause early onset renal failure in X-linked disease are associated with lenticonus and retinopathy [14].

The number of different published *COL4A5* variants has increased from 176 in 1997 [26], to 520 by 2010 [27], and 1200 in 2015. Many more pathogenic variants have been reported for *COL4A5* than for *COL4A3* and *COL4A4*. There was some bias in the types of mutations reported here (deletions and insertions were more common for *COL4A5)* probably because of the detection techniques.

The major challenge for the future is to encourage all diagnostic laboratories including those that are commercially-based, to submit their variants to a database, together with accurate pathogenicity assessments and as much clinical information as possible. The classification of some so- called benign variants will be revised. We will understand better genotype-phenotype correlations for heterozygous *COL4A3* and *COL4A4* mutations in Thin basement membrane nephropathy, and any genetic distinctions from variants that cause autosomal dominant Alport syndrome. In addition we will better understand the consequences of biallelic, triallelic and digenic *COL4* mutations [25], and possibly why some heterozygous *COL4* mutations are associated with proteinuria.

Increasingly the value of sharing variants and pathogenicity assessments is realised. Some countries allow all variants identified in public laboratories to be shared in public databases without specific assent, and IRBs internationally are finding that the public benefit of sharing outweighs any small adverse risk. Our study exemplifies the advantages of sharing variants in a rare disease.
